# Dynamics of microflow at the plasma–liquid interface

**DOI:** 10.1038/s41598-022-20693-8

**Published:** 2022-10-05

**Authors:** Lucia Kuthanová, Tomáš Hoder

**Affiliations:** Department of Physical Electronics, Masaryk University, Kotlářská 2, 61137 Brno, Czech Republic

**Keywords:** Fluid dynamics, Plasma physics

## Abstract

We study the interaction of microplasma with viscous liquid in a narrow gap. The reduced surface tension and viscosity of the liquid droplet from local plasma-heating induce a radial fingering. The introduced methodology enables spatially and temporally resolved quantification of dissipated power density and of resulting velocity of the advancing plasma–liquid interface. For two plasma power scenarios, we demonstrate how the irregular distribution of the two parameters leads to microflow, interface stretching, and to primary droplet fragmentation via capillary instability and end pinching.

## Introduction

The dynamics of liquids flowing in microscopic volumes is very important to many biological and technological systems, including microfluidics, electrowetting, nutrition distribution in living organisms or coating processes. The viscosity and the surface tension of the transferred liquid are usually among the main parameters determining the microflow and are investigated in many scenarios. For example, the viscous fingering of liquids injected in a narrow gap of Hele-Shaw cell^1^ is studied for both technological^2^ and fundamental reasons^3^. Droplet transport in narrow channels is important for digital microfluidics^4^ as well as in physical chemistry^5^ and the detailed knowledge about thin films spreading on silicon wafers can be utilised in coating applications^6,7^.

External force fields are often used to initiate or manipulate the microscopic flow via hydrodynamic instabilities for various purposes. Electrical charging may lead to destabilisation of toroidal droplets according to the Saffman–Taylor theory^8^ while in tapered Hele-Shaw cells^9^ or electrically manipulated electrolytes^10^ the fingering pattern can be controlled. In some cases, a combination of the applied external forces results in the liquid microflow instability, too^6^. In direct reference to the crucial role of the liquid viscosity and surface tension, these fingering patterns are in many cases connected to Marangoni effect or Saffman–Taylor instability, depending on the arrangement, parameters of the fluid, of the neighbouring materials and on the complexity of the external force.

Moreover, it was observed that a similar fingering instability in a narrow gap can also be induced by atmospheric plasma^11^, with direct implications for recently intensively studied phenomena at plasma–liquid interface^12,13^ and its use in microfluidics or biomedical applications^14–17^. Due to its complex electrical and thermal action, atmospheric plasma shows a possible way how to locally and in a scalable way change the local physicochemical properties of the contacting liquid or to manipulate its flow via altered wettability of close surfaces. The plasma can even stabilize an otherwise unstable liquid interface^18^. For such utilization of the microplasma a local, microscopic understanding of the dynamics at plasma–liquid interface is necessary.

In this paper, we investigate the atmospheric microplasma interaction with droplet of viscous liquid in Hele-Shaw cell. We propose and utilise a new methodology, which reveals a quantified spatially resolved distribution of the original cause of microscopic flow, the local power density dissipated in the droplet-surrounding plasma. Furthermore, we quantify its resulting effects via the local velocity field of the moving plasma–liquid interface. We show how the irregularities of these parameters along the interface lead to the initiation of the microflow, fingering process and to liquid channels fragmentation to secondary droplets via capillary instability. In this way, we present a detailed insight into local properties of the plasma-initiated microflow.

## Experimental setup

**Figure 1:**
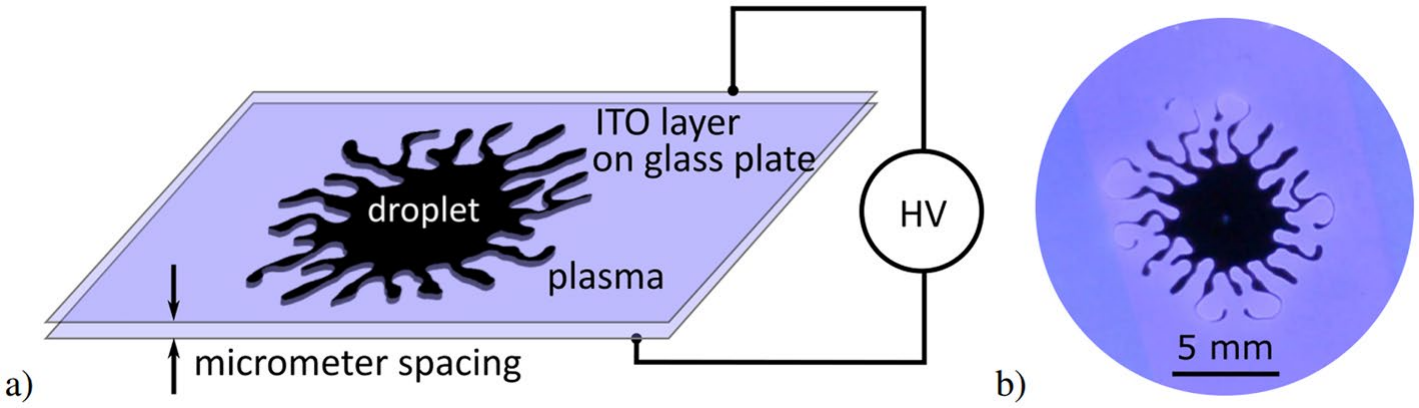
(**a**) The simplified schematic of the experimental setup for plasma generation in Hele-Shaw cell containing the viscous droplet and (**b**) the photograph of the viscous liquid fingering pattern (dark) induced by the surrounding plasma (bluish).

The scheme of the experimental arrangement and the photograph of the investigated hydrodynamic instability is shown in Fig. 1. The Hele-Shaw cell consists of two glass sheets (relative permittivity $\epsilon _\mathrm {r} = 4.6$) with thin layers of ITO ($320\,$ nm) deposited on outer sides of them, acting as transparent electrodes. The plasma of dielectric barrier discharge is generated in ambient air in the gap (width $b= 100\,\upmu$m) between the glasses, where the squeezed droplet ($8 \upmu$l) is also placed. As a viscous liquid, polydimethylsiloxane oil with relative permittivity $\epsilon _\mathrm {r} = 2.8$, density $\rho = 970$ kg/m^3^, kinematic viscosity of $\nu =100$ mm^2^/s and surface tension $\sigma =20.9$ mN/m, all at 25 °C, was chosen. These values are temperature dependent and in the calculations further in the paper their corresponding temperature-adjusted values were used.

The discharge was powered by AC high voltage generator providing sinusoidal voltage operating at frequency $f=12.5$ kHz, with maximum voltage amplitude $U_{\mathrm {amp}}=10$ kV. Electrical current and voltage measurements were performed with current probe (Tektronix CT-2) and voltage probe (Tektronix P6015A), both connected to a high-resolution and high-sampling rate oscilloscope (Keysight DSO-S 204A 2 GHz 20 GSa). Imaging of the discharge was performed with an ICCD camera (PI-MAX3 1024RB-25-FG-43) mounted above the cell. The ICCD camera and oscilloscope measurements were both synchronized with the discharge.

## Results and discussion

If confined in the Hele-Shaw cell and surrounded by an atmospheric plasma, a droplet of viscous liquid can undergo an interfacial instability, viscous fingering (see Fig. 1b), and end up fragmented to many secondary droplets, as was shown in ^19^. Such behaviour is dependent on many parameters, such as gap spacing, voltage applied for plasma generation, or fluid properties, mainly viscosity and surface tension. However, the fluid properties are dependent on temperature and the temperature can be locally and temporarily elevated by plasma presence. While the effect of the surface tension thermal dependence was discussed already in^20^, only recently it was shown that the microplasma heating is affecting the liquid flow also via thermal modification of the viscosity of the liquid^19^. In this approach, it is assumed that the temperature rises linearly with the plasma-driving applied voltage amplitude^19,21^. The methodology of determining the slope of the $\mathrm {d}T/\mathrm {d}U$ was described in^19^ and the results were discussed in terms of the maximum temperature $T_\mathrm {max}$ reached for maximum used voltage $U_\mathrm {amp}=10$ kV. For the size of the droplet 8 μl the $T_\mathrm {max}$ is in the range 325–391 K.

**Figure 2:**
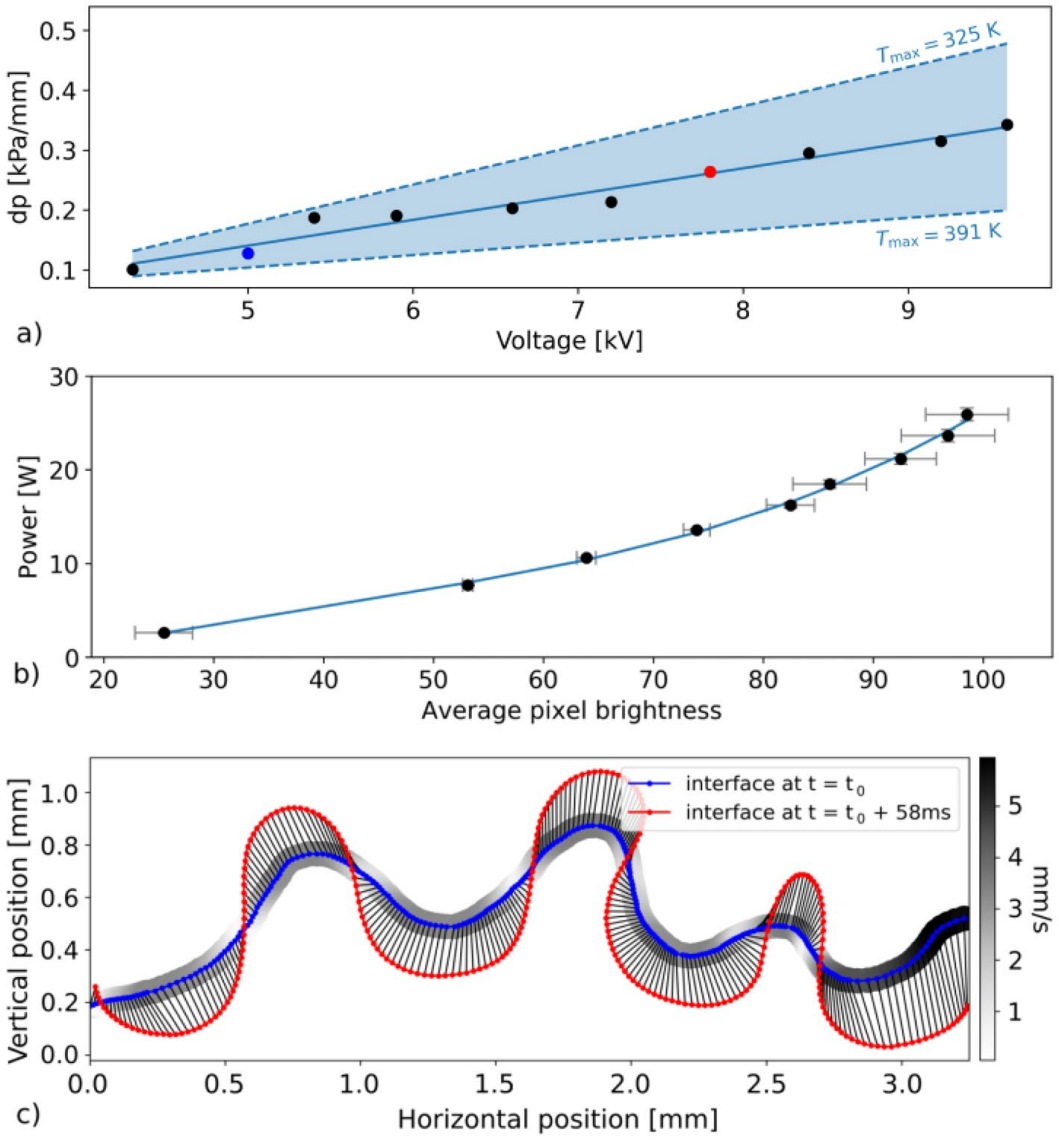
The elements of the developed methodology. (**a**) The accurate determination of the pressure gradient responsible for the microflow as obtained from Darcy’s law with temperature dependent viscosity. (**b**) The calibration curve showing the power in dependency on the pixel brightness of the ICCD images. (**c**) Determination of the local velocity of the plasma–liquid interface section.

The viscous flow in narrow gaps, as in our experiment, is described by Darcy’s law $u = \frac{-b^2}{12\nu \rho } \nabla p$. Here, *u* is the average velocity, *b* the sheet spacing, $\nu$ and $\rho$ are the liquid viscosity and density and $\nabla p$ is the macroscopic pressure gradient responsible for the flow. Thanks to the detailed analysis of video frames and with the help of the model developed for description of the fingering instability wavelength in^19^, we were able to evaluate the pressure gradient accurately, taking the temperature dependence of viscosity $\nu (T)$ and density $\rho (T)$ into account. It is plotted for a range of applied voltages in Fig. 2a) and it clearly exhibits a linear relation. The dashed blue lines are fits corresponding to the upper and lower extremes of temperature range resulting from^19^, representing a confidence band, and the data points are respective temperature-averaged values. Two different voltage amplitudes (denoted by blue 5 kV and red 7.8 kV) will be investigated in detail later in the text as two different voltage scenarios. Each time a temperature dependent parameter will be calculated, its range for ($T_\mathrm {max} = 325$ K − $T_\mathrm {max} =$ 391 K) will be stated.

While before, only global parameters have been assumed for the whole system based on applied voltage and overall power consumption, now we have pursued to determine it more precisely, with spatial and temporal resolution. To be able to localize and quantify the source of the local temperature rise, the local dissipated power, we have performed a calibration of the spatially resolved detected optical emission intensity of the plasma with the global power parameter. The optical emission intensity of the plasma is generally a measure of a number of emitting radiative states excited dominantly by the electrons accelerated by the applied voltage. The emission intensity is therefore a good candidate to be a local measure of the dissipated electric power. The emission intensity was evaluated as an average brightness of each plasma-displaying pixel in the ICCD video frame. The power was determined from the electrical measurements according to the equation $P(t) = \frac{1}{\tau }\oint U I dt$. Here, *P* is the power dissipated in the plasma, $\tau$ denotes the period of the applied voltage waveform, *U* is the measured voltage and *I* the measured current. To suppress stochasticity of each period, 130 consecutive periods were averaged each time.

**Figure 3:**
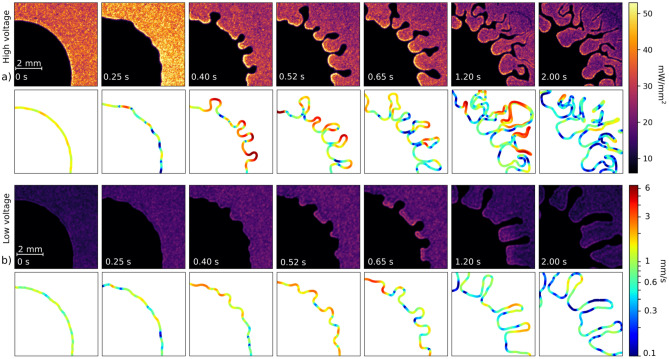
Simultaneous visualisation of the two-dimensional development of the local dissipated power density (first and third row, upper scale bar) and velocity field (second and fourth row, lower scale bar) at the interface for (**a**) high (7.8 kV) and (**b**) low (5 kV) voltage scenarios. The scales for power density and velocity are common for both voltage scenarios.

**Figure 4:**
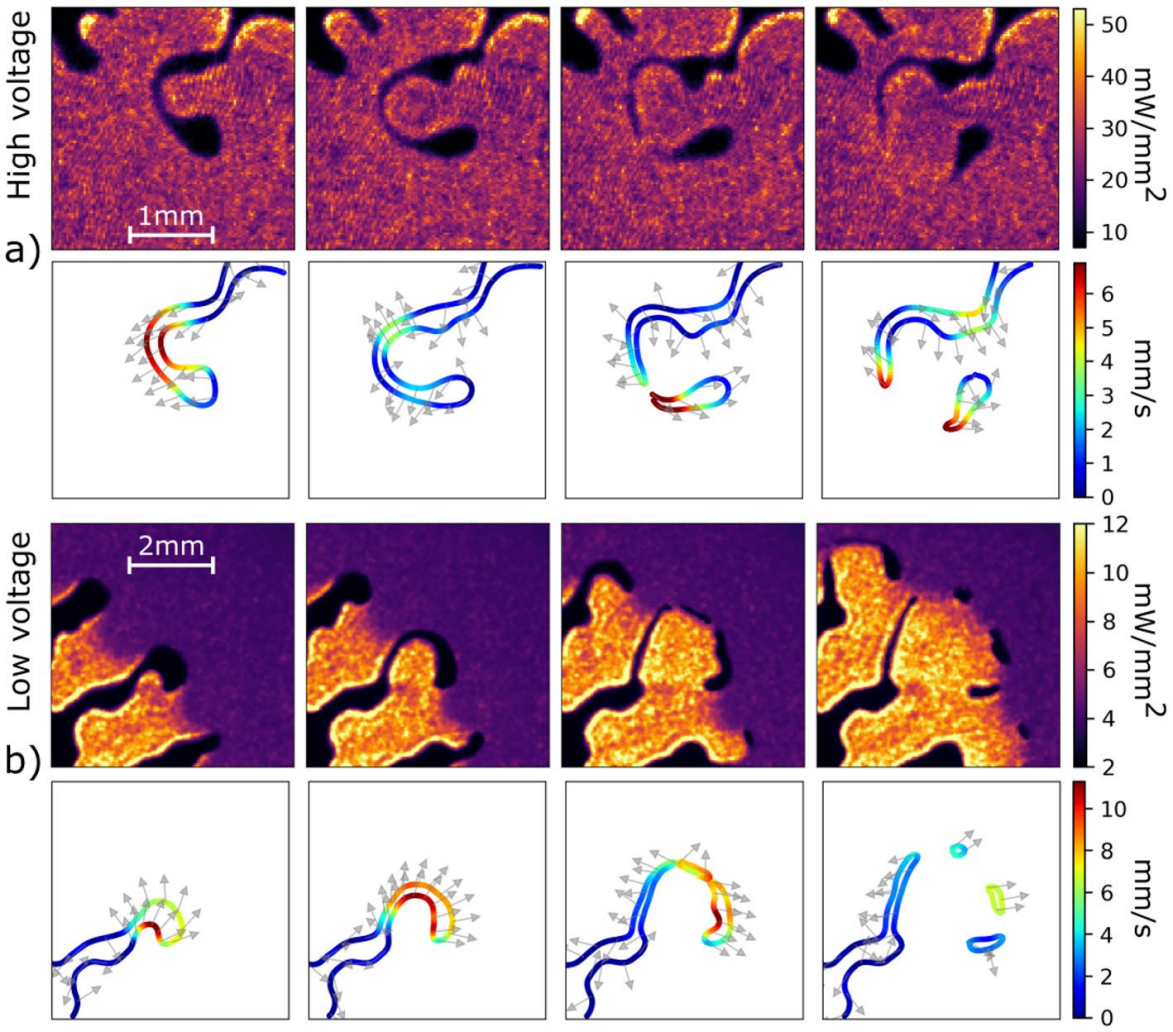
Secondary droplets generation in (**a**) high and (**b**) low voltage plasma. The high voltage sequence starts 1.4 s after the voltage switch-on, low voltage sequence after 3.5 s. The time step between the consecutive frames is 58 ms.

The calibration itself was then performed by comparing the averaged pixel brightness development to the measured power development over a wide range of applied voltages. In a reverse way, we have then recomputed the brightness scale in the two-dimensional pictures recorded by the ICCD camera to the power density scaled maps. The calibration curve is shown in Fig. 2b) and the results of such procedure will be shown in Figs. 3 and 4.

For the description of the ongoing microflow, the local velocity vector and its development is another key parameter. Using a self-developed semi-automatised plasma–liquid interface recognition software, we have analysed the recorded ICCD data and evaluated changes between consecutive frames. The algorithm started by detecting the interface in individual frames. The obtained 2D image of interface outlines was then converted into a curve in *x*, *y* coordinates, more suitable for further evaluation. The shift between corresponding elements of the curves in consecutive frames was determined and divided by the frame duration. In such way, the velocity vector field for each frame was determined, see the example in Fig. 2c).

The above described methodologies were applied to the investigated plasma-droplet system for two different power scenarios (with voltage amplitudes 5 kV and 7.8 kV) and the results are shown in Fig. 3. These results represent the typical cases of system evolutions at low voltage and high voltage (already highlighted in colour in Fig. 2a).

The dissipated plasma-power density maps in Fig. 3, first and third row, clearly reveal that the power density is at much higher levels for higher voltage amplitude, with the peak values being up to 50$_\mathrm {H}$ mW/mm^2^ and 25$_\mathrm {L}$ mW/mm^2^ in the high and low voltage scenarios, respectively (see the notation in subscript). In general, it results in faster interfacial changes for the high voltage scenario. The distribution of the dissipated power is visibly uneven in most of the images due to different local plasma activity. The peak values are in both cases located at the tips of the advancing plasma fingers. Apparently, the driving force for the fingering process, the plasma-induced heating, originates from these areas, while the resulting fast advancing of the liquid channels' tips is well visible from the velocity field graph (see in Fig. 3, second and the fourth row). The liquid is driven from the hot areas into the oil channels, as it is allowed by the lowered local viscosity. The opposite localisation of the power and velocity maximums in early, decisive stages is pronounced in both scenarios, best visible in the high voltage one at 400 ms. In later stages, the fingering velocity decreases and the velocity distribution along the finger interface is more stochastic, as the power density is distributed more homogeneously along the interface.

The highest velocities of the moving interface were achieved in the high voltage scenario. The local velocity peaks at 6.9$_\mathrm {H}$ mm/s and 4.1$_\mathrm {L}$ mm/s for high and low voltage scenarios, respectively. With the limitations in locality and spatial scale approaching the scale of the *b* parameter, we may only estimate the local pressure gradient from these velocities applying the Darcy’s law. It results in the pressure gradients peaking at (0.53–0.24)$_\mathrm {H}$ kPa/mm and (0.36–0.21)$_\mathrm {L}$ kPa/mm. The capillary number $\mathrm {Ca} = \frac{\nu \rho v}{\sigma }$, which represents the relation between the viscous drag forces and surface tension forces, and is frequently used to classify different modes of hydrodynamic behavior^22,23^, was calculated to be (0.0226–0.0120)$_\mathrm {H}$ and (0.0151–0.0095)$_\mathrm {L}$. Reynolds numbers $\mathrm {Re} = \frac{v L}{\nu }$ for both high and low voltage scenarios, (0.0100–0.0208)$_\mathrm {H}$ and (0.0052–0.0087)$_\mathrm {L}$, are very low, proving that inertia plays only a very small part in determining the local microscopic motion, which is dominated by viscous forces.

In both scenarios, the microflow leads to very intense interface stretching. In high voltage case, in less than 2 s the rapid prolongation of the fingers resulted in such drastic thinning of the oil channel that it approached the dimension of the glass sheets spacing *b* and fragmented itself via capillary effects into several secondary droplets^24^. The more detailed results on channel fragmentation and droplet pinch-off are shown in Fig. 4. The sequences of the video frames suggest that the extreme thinning of the channel and its fragmentation starts when a “bay” of plasma enclosed by a single oil channel from multiple sides is formed. This enclosed plasma starts to push towards the oil, just as the fingers of primary instability in Fig. 3 did. The oil channel reacts by shifting itself outwards, thus increasing the “bay” radius and its own length. Unlike the primary plasma fingers, the enclosed plasma acts asymmetrically and onto a limited liquid volume in the thinning channel. As a result, the channel stretches beyond the limit and the fragmentation and secondary droplet pinch-off occur. The observed sequence is very similar to phenomena investigated in rotating Hele-Shaw cells without plasma^25,26^.

Based on the study of tens of such channel fragmentations, the average channel width in the last moment before the fragmentation was found to be $(110 \pm 10)_\mathrm {H} \, \upmu$m and $(260 \pm 50)_\mathrm {L} \, \upmu$m, for high and low power, respectively. The average diameter of the secondary droplets, the number of the droplets created from one channel and their density is $(160 \pm 10)_\mathrm {H} \,\upmu$m, $(11.80 \pm 7.25)$ $_\mathrm {H}$ droplets and $(3.93 \pm 0.49)$ $_\mathrm {H}$ droplets/mm for high voltage and $(430 \pm 70)_\mathrm {L}\, \upmu$m, $(2.83 \pm 0.90)$ $_\mathrm {L}$ droplets and $(1.00 \pm 0.24)$ $_\mathrm {L}$ droplets/mm for low voltage scenario.

The maximum local velocity of the interface of fragmenting oil channel reached approx. 7$_\mathrm {H}$ and 11$_\mathrm {L}$ mm/s for the two scenarios. While in high voltage case, this value is close to those observed before the fragmentation, in low voltage this velocity value significantly exceeds the maximum velocities reached during the initial stages of the fingering process. The corresponding Darcy’s pressure gradient estimates for channel fragmentation are (0.54–0.25)$_\mathrm {H}$ and (0.98–0.56)$_\mathrm {L}$ kPa/mm.

**Figure 5:**
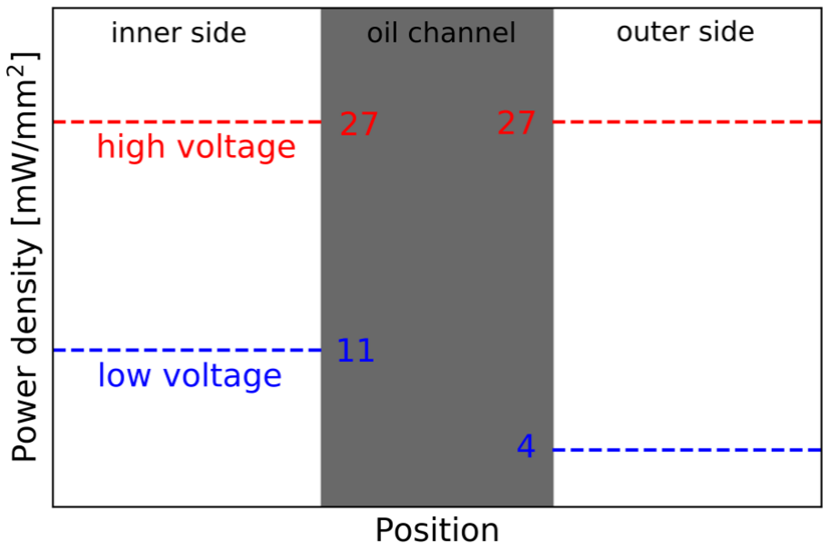
Schematic depiction of the average power densities on the two sides of the oil channel during the fragmentation. Note that the power density is the same on both sides of the channel in case of high voltage, but asymmetrical (and much lower) in low voltage case.

Above mentioned differences between the fragmentation behaviour in low versus in high voltage can be attributed to the asymmetrical power density on the two sides of the channel in the case of the low power scenario, see Fig. 4 and schematic depiction of the power densities on the two sides of the oil channel in Fig. 5. While on the inner side (closer to the primary droplet centre) of the channel the power density rises up to above 10 mW/mm^2^, on the outer side it is as low as only few units of mW/mm^2^. The plasma heating is therefore much more intense from one side of the channel. In the high power scenario presented here, comparable power densities of few tens of mW/mm^2^ were detected on both sides of the channel. Although the asymmetrical conditions were not always present for low voltage and absent for high voltage, in low voltage case they were necessary for secondary droplet pinch-off.

We propose that two mechanisms contribute to the channel fragmentation under given conditions, the capillary-wave instability and the end pinching mechanism, similarly as introduced for the free drops at low Reynolds numbers in^27–29^. While in high voltage case the fragmentation shows high periodicity, high density, smaller secondary droplets diameters and lower pressure gradients at the very moment of breakup, the low voltage scenario results in fewer bigger secondary droplets and comparably much higher pressure gradient at the breakup moment (see Fig. 4). The asymmetric heating of the channel in the low voltage scenario is suspected to be the cause for the end pinching mechanism to be dominant over the capillary-wave instability in this case. The capillary number for breakup conditions at low voltage case reaches almost three times higher values ((0.0405–0.0256)$_\mathrm {L}$) than during the unfragmented oil channel propagation. The aspect ratio prior to the breakup *L*/*d* = (17 ± 8)$_\mathrm {L}$^30^ further classifies the breakup^31,32^. For high voltage breakup it was *L*/*d* = (56 ± 29)$_\mathrm {H}$.

## Conclusion

In summary, the presented simultaneous quantification of the local dissipated plasma-power density and resulting velocity field of the plasma–liquid interface is a powerful methodology to study the very causes and consequences of plasma–liquid interaction in narrow gaps or porous media. The quantification of dissipated plasma-power directly at the liquid interface and the subsequent liquid mass displacement are of crucial importance in emerging applications of plasmas in microfluidics and medicine^12,14,15^. The presented system and the developed methodology also offer a new opportunity for systematic study of the liquid channel breakup and droplet pinch-off due to a complex external force in 2D geometry of Hele-Shaw cell at low Reynolds numbers and high viscosity contrast, an issue of general importance^33–35^.

## Data Availability

The datasets used and analysed during the current study are available from the corresponding author on reasonable request.
